# Up-regulation of S100P predicts the poor long-term survival and construction of prognostic signature for survival and immunotherapy in patients with pancreatic cancer

**DOI:** 10.1080/21655979.2021.1992331

**Published:** 2021-10-27

**Authors:** Wenbo Zou, Lincheng Li, Zizheng Wang, Nan Jiang, Fei Wang, Minggen Hu, Rong Liu

**Affiliations:** a Medical School of Chinese PLA, Beijing, China; b Faculty of Hepato-Pancreato-Biliary Surgery, The First Medical Center of Chinese People’s Liberation Army (PLA) General Hospital, Beijing, China; c Key Laboratory of Digital Hepetobiliary Surgery, PLA, Beijing, China

**Keywords:** Pancreatic cancer, immune-related gene, prognosis, immune cell infiltration, tumor mutation burden

## Abstract

Pancreatic cancer is associated with a high mortality rate, and the prognosis is positively related to immune status. In this study, we constructed a prognostic signature from survival- and immune-related genes (IRGs) to guide treatment and assess prognosis of patients with pancreatic cancer. The transcriptomic data were obtained from The Cancer Genome Atlas (TCGA) database, and IRGs were extracted from the ImmPort database. Univariate and LASSO regression analysis were used to obtain survival-related IRGs. Finally, the prognostic signature was constructed using multivariate regression analysis. The laboratory experiments were conducted to verify the key IRG expression. Immune cells infiltration was analyzed using the CIBERSORT algorithm and TIMER database. Prognostic signature containing four IRGs (ADA2, TLR1, PTPN6, S100P) was constructed with good predictive performance; in particular, S100P played a significant role in the immune microenvironment, and tumorigenesis of pancreatic cancer. Moreover, we found that CD8^+^ T cell and activated CD4^+^ memory T cell tumor infiltration was lower in the high-risk group, while high-risk score correlated positively with higher tumor mutational burden, and the higher half inhibitory centration 50 of chemotherapeutic agents Docetaxel and Sunitinib. In summary, this study identified and constructed an immune-related prognostic signature that can predict overall survival, besides suggests that S100P was a novel immune-related biomarker. We hope that this signature will aid the identification of new biomarkers for the individualized immunotherapy of pancreatic cancer.

## 1. Introduction

Pancreatic cancers have one of the highest mortality rates, with an average 5-year survival rate of approximately 10% in the United States [1]. A lack of obvious early symptoms and difficult diagnosis lead to poor treatment efficacy and prognosis, thus increasing mortality. Radical surgery and chemotherapy are currently the main treatment strategies for pancreatic cancer and the development of various comprehensive treatments has gradually increased the overall survival (OS) of patients [2,3]; however, few patients are suitable for surgical treatment due to the existence of distant metastasis or local invasion, and resistance to chemotherapy is inevitable [4]. Immunotherapy has become increasingly popular in recent years and has revolutionized oncotherapy since it can mobilize the patient’s immune system to enhance its antitumor abilities [5]. For example, recent approaches targeting the inhibition of programmed cell death 1 (PD-1) or programmed cell death ligand 1 (PD-L1) have been successfully used to treat various tumors [6–8].

The abnormal expression of immune-related genes (IRGs) is closely related to the progression of malignant tumors and offers a new perspective for exploring prognostic biomarkers [9]. In addition, the prognostic efficacy of IRGs is closely related to the tumor immune microenvironment (TIME) and studies have reported that an IRG-immunocyte-TIME interaction network plays a crucial role in tumorigenesis and progression [10,11]. Various prognostic signatures based on IRGs have recently been constructed to predict the prognosis of patients with cancer and have proven to effectively predict OS and aid the exploration of novel biomarkers [12–14]. Recently, few reliable prognostic signatures have been reported in patients with pancreatic cancer and the mechanisms underlying IRGs have been confirmed. Wu et al. found that three IRGs CKLF, ERAP2, and EREG showed distinct relationships with pancreatic cancer patients’ survival [15], Wang et al. Chen et al. and Zhang et al. identified some IRGs which was highly related with long-term survival of patients with pancreatic cancer respectively [16–18]. However, most current prediction signatures focus only on predicting long-term survival and tumor progression in pancreatic cancer patients. But more importantly, to our knowledge, tumor immune microenvironment plays its important role in immunotherapy, it is therefore significant to identify a strong prognostic signature based on IRGs that could affect the tumor immune status and guide the precise immunotherapy of pancreatic cancer in the future. Meanwhile, the role of more IRGs in the malignant progression of pancreatic cancer still needs to be further identified and revealed.

In this study, we constructed a prognostic signature that can independently predict tumor prognosis of pancreatic cancer patients when combined with clinicopathological characteristics. Notably, we applied the prognostic signature to predict chemotherapeutics efficacy for precise which may aid personal therapy in future. In addition, we analyzed and validated S100P, a key IRG via bioinformatics analysis and laboratory experiment involved in quantitative real-time PCR and immunohistochemistry. To sum up, we explored potential regulatory mechanisms that highlight the strong relationships between IRGs and pancreatic cancer. Hopefully, this signature will aid the identification of new biomarkers for the individualized immunotherapy of pancreatic cancer.

## 2. Materials and methods

### 2.1. Data download and preprocessing

We obtained the transcriptomic data and clinicopathological characteristics of 178 pancreatic cancer tissues and 4 adjacent normal tissues from TCGA database for preprocessing. A total of 2483 immune-related genes (IRGs) were extracted from the Immport database (https://immport.niaid.nih.gov) [19]. The mRNA matrix annotated as ‘protein-coding’ was extracted for subsequent screening.

### 2.2. Differentially expressed genes (DEGs) and functional enrichment analysis

DEGs were screened based on the mRNA matrix using the ‘limma’ package [20] with thresholds of adjusted *p* value < 0.05 and |log2 fold change (FC)| > 1. The IRGs were then intersected with DEGs to obtain differentially expressed IRGs. To explore the potential functions of these IRGs, we performed Gene Ontology (GO) and Kyoto Encyclopedia of Genes and Genomes (KEGG) enrichment analyses using the ‘clusterProfiler’ [21] and ‘GOplot’ packages [22]. A protein-protein interaction (PPI) network was constructed from the IRGs using the STRING online database (https://string-db.org/) [23], with a medium confidence threshold of 0.4.

### 2.3. Construction and validation of the immune-related prognostic signature

We combined survival data with IRGs expression levels and conducted survival analysis to select the independently survival-related IRGs. The ‘survival’ package was used to perform univariate regression analysis to screen survival-related IRGs, which were filtered using LASSO regression in the ‘glmnet’ package [24]. Finally, a prognostic signature containing four significantly survival-related IRGs was constructed using multivariate regression analysis. Risk scores were calculated from the regression coefficient and expression levels of the four IRGs and then all patients were divided into high- and low-risk groups based on the median risk score. To verify the prediction accuracy of the signature, we calculated the area under the curve (AUC) of the receiver operating characteristic (ROC) using the ‘timeROC’ package. A Kaplan-Meier (KM) survival curve was produced using the ‘survival’ and ‘survminer’ packages to describe the predictive power of the indicators. We also analyzed the correlation between the signature and clinicopathological characteristics, and investigated the independent predictive capability of our signature using Cox regression analysis with a threshold of *p* < 0.05. Finally, Gene set enrichment analysis (GSEA) was conducted to reveal the mechanism and pathways underlying the prognostic signature [25].

### 2.4. Tissue collection and cell culture

Pancreatic cancer and normal tissues were prospective collected from the Chinese PLA general Hospital. This study was approved by the ethics committee of the Chinese PLA general Hospital. Written informed consent was obtained from all patients. BxPC-3, and SW1990 pancreatic cancer cells and HPDE6-C7 normal pancreas cells were purchased from the American Type Culture Collection (ATCC). The BxPC-3, SW1990, and HPDE6-C7 were incubated in RPMI-1640 medium (Solarbio, Beijing, China). All the mediums were supplemented with 10% fetal bovine serum (FBS, Gbico, USA), 100 units/ml penicillin G, and 100 ug/ml streptomycin, and were cultured at 37°C in a damp incubator, which was supplemented with 5% CO_2_.

### 2.5. Expressed validation and potential mechanisms exploration of S100P

We downloaded the transcription factor (TF) dataset from the Cistrome Cancer database [26] and obtained differentially expressed TFs based on the DEGs, with thresholds of adj. *p* < 0.05 and |logFC| > 1. Correlation analysis was then performed between the differentially expressed TFs and the four IRGs, with a minimal 0.4 coefficient and *p* < 0.05. After analyzing the DEGs of two transcriptomic data (GSE32676, GSE28735) from the GEO database, we found that S100P was a common DEG that overlapped with a survival-related gene screened from the TCGA database. Therefore, we conducted bioinformatic analyses to explore the potential biological role of S100P in tumor immunity.

The TISIDB database was used to produce the KM survival curve and the correlations between S100P expression and immune inhibitors [27], while the GEPIA database that integrate the expression of genes in TCGA and GTEx data [28], was used to present the KM survival curve, and to analyze the differential expression level of S100P. The TIMER database was used to explore the correlation between S100P and tumor-infiltrating cells, since it is often used to systematically evaluate the relationship between immune cells and target genes in cancers [29]. Notably, quantitative real-time PCR (qRT-PCR) were performed to validate the differential expression level of S100P: TRIzol reagent (Ambion) was used to extracted total RNA; NanoPhotometer^®^ C40 Touch (IMPLEN) was used to assess the RNA purity based on the ratio of OD260/280 and 260/230; Eppendorf Mastercycler^®^ was used to perform reverse transcription of qualified RNA to single-stranded complementary DNA according to the manufacturer’s instructions; StepOnePlus^TM^ Real-Time PCR instrument was used to implement real-time quantification; 18S rDNA was used as internal reference; calculation of the relative expressions used the 2^−ΔΔCt^ method. Primers sequences of S100P and 18S rDNA were shown in (Table 1). Finally, the immunohistochemical image of S100P were downloaded from the Human Protein Atlas (HPA) database for comparing differential expression of S100P between the tumor and normal tissues in protein expression levels.

**Table 1.** Primers used for quantitative real-time PCR

GeneName	Direction	Sequences (5ʹ–3ʹ)
S100P	Forward	AAGGATGCCGTGGATAAATTGC
S100P	Reverse	ACACGATGAACTCACTGAAGTC
h18S	Forward	AACCCGTTGAACCCCATT
h18S	Reverse	CCATCCAATCGGTAGTAGCG

### 2.6. Development and validation of the nomogram

The nomogram combining the IRGs signature with clinicopathologic characteristics was developed to predict the 1-, 2- and 3-year survival of patients with pancreatic cancer, and calibration curves were generated to evaluate the performance of the nomogram. All the analyses were performed using the ‘rms’ and ‘foreign’ packages.

### 2.7. Association of risk score with immune cell infiltration, chemotherapeutics efficacy and tumor mutation burden (TMB)

To analyze differences in immune cell infiltration between risk groups, CIBERSORT, an algorithm that can filter meaningful samples [30] was used with *p* < 0.05 and perm was set to 100. it can calculate the distribution of 22 immune cell types in each sample and analyzed the differences in immune cells across risk groups. Subsequently, the correlation between the four key IRGs and common immune cells was also explored in the TIMER database. Then we evaluated the power of risk score to predict chemotherapeutics efficacy in PC, and calculated the half inhibitory centration (IC50) difference of several chemotherapeutics in high- and low- risk groups using the Wilcoxon signed-rank test and ‘pRRophetic’ package [31]. Finally, we downloaded TMB data from TCGA database, calculated the TMB scores for each patient, and assessed the association between TMB score and risk score.

### 2.8. Statistical analysis

R version 4.0.2 software, its resource packages, and GraphPad Prism 8.0 software were used for all statistical analyses and to plot relevant visualizations. The statistical significance of all tests performed in this study was determined as a two-sided *p* value of < 0.05.

## 3. Results

### 3.1. Differentially expressed analysis

**Figure 1.**
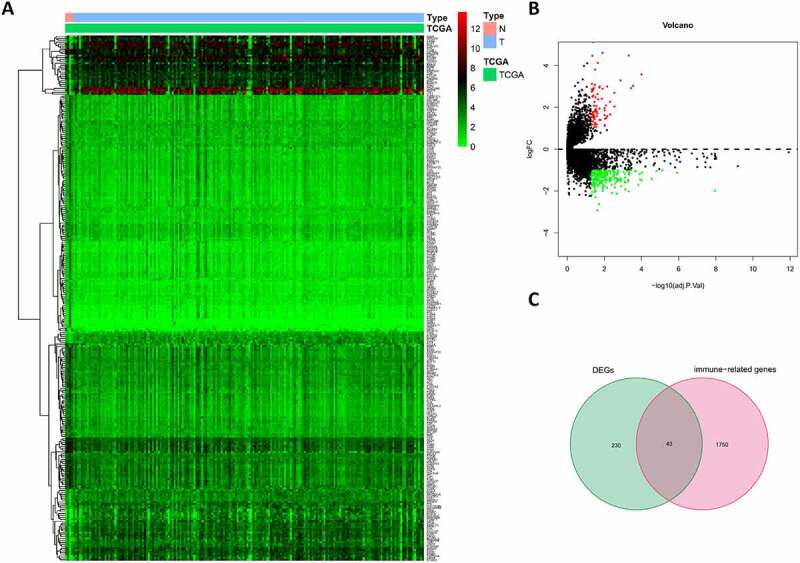
Differentially expressed analysis. (a) Heatmap of significant DEGs in pancreatic cancer. (b) Volcano plot of DEGs. (c) The Venn diagram of the intersection between DEGs

By screening the expression of 19645 mRNAs in tumor and peritumor tissues, we identified 273 DEGs, of which 71 were up-regulated and 202 were down-regulated (Figure 1(a,b)). Intersecting the DEGs and IRGs revealed 43 differentially expressed IRGs (Figure 1(c)). As expected, GO and KEGG pathway analysis showed that the functions of these IRGs correlated significantly with immune cell infiltration and the immune response (Figure S1(a–c)), while the PPI network revealed associations between these 43 IRGs (Figure S1(d)).

### 3.2. Construction and validation of the prognostic signature

**Figure 2.**
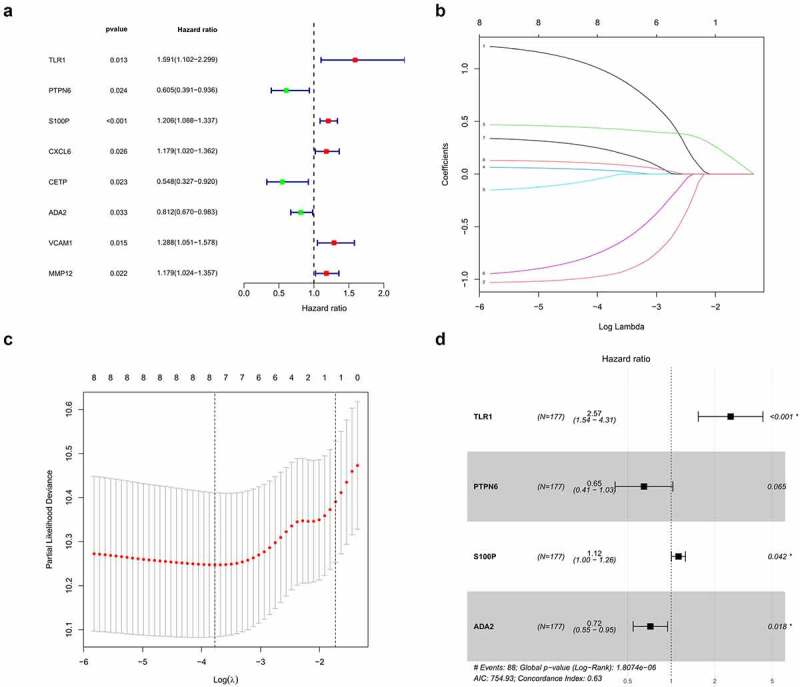
Construction of the prognostic signature. (a) Univariate regression analysis. (b,c) LASSO regression analysis (d) Multivariate regression analysis

Univariate and LASSO regression analyses identified eight survival-related IRGs (*p* < 0.05, Figure 2(a–c)), from which, an optimal signature of four significant IRGs was constructed using multivariate cox regression (Figure 2(d)). Risk scores were calculated from four IRGs coefficients and expressions as follows $\begin{array}{r} risk\;score=(0.11609{)}^{*}S100P+(-0.43342{)}^{*}\\ PTPN6+(0.94539{)}^{*}\\ TLR1+(-0.33095{)}^{*}ADA2 \end{array}$

**Figure 3.**
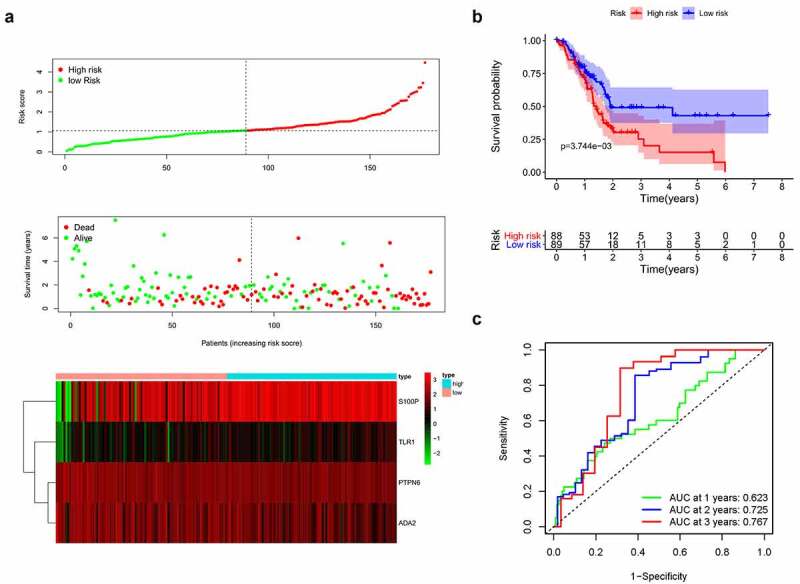
(a) Survival condition plots and heatmap of four IRGs. (b) Kaplan-Meier survival curve. (c) Time-dependent ROC curves used to predict OS at 1, 3, and 5 years

Based on the median risk score, we divided patients into high- and low-risk groups (*n* = 88 and 89, respectively). Survival condition plots showed that the high-risk group had a higher mortality than the low-risk group and revealed the differential expression patterns of the four IRGs (Figure 3(a)). Moreover, the KM survival curve indicated that the high-risk group had a poorer prognosis and a shorter OS than the low-risk group (*p < *0.01, Figure 3(b) and Table S1). To verify the predictive accuracy of this signature, we calculated the AUC at 1, 2, and 3 years (0.623, 0.725, and 0.767, respectively), which suggests that our signature has predictive accuracy (Figure 3(c)).

### 3.3. Potential immune-related mechanism of S100P

**Figure 4.**
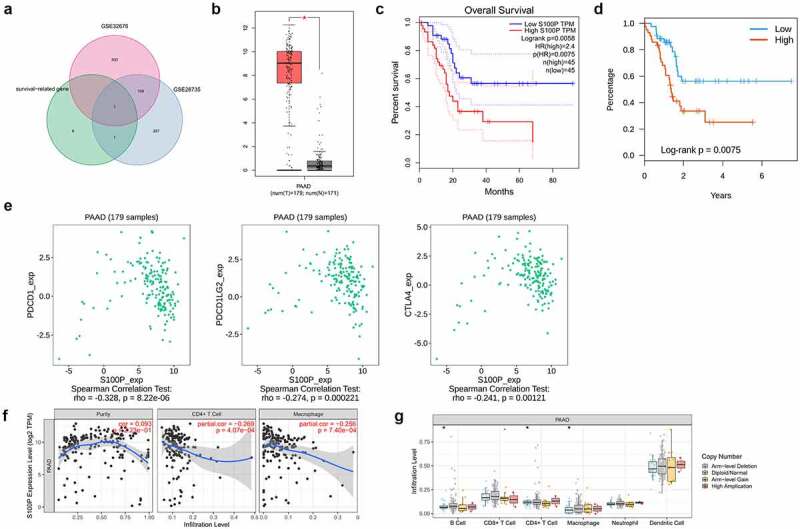
Mechanism analysis of S100P. (a) Venn diagram for the intersections of IRGs and data from the GEO database. (b) Differentially expressed analysis of S100P in GEPIA database. (c,d) Survival analysis of S100P. (e) The relationship between the common immune-inhibiter and S100P. (f) The correlation of immune cell infiltration with the expression level of S100P. (g) Copy number variation analysis

By comparing the survival-related IRGs and the DEGs of two other transcriptomic data from the GEO database, we identified S100P as a common DEG (Figure 4(a)). In addition, we obtained four differentially expressed TFs (CIITA, FLI1, KLF5, SPDEF; *p* < 0.001, Table 2), of which two (KLF5, SPDEF) were strongly positively correlated with S100P (Table 3), suggesting that S100P could be a key IRG with an important role in pancreatic cancer.

**Table 2.** Differentially expressed TFs

TF	logFC	AveExpression	p value	adj.p value
CIITA	−1.4903896	1.782997979	0.0000163	0.00195858
FLI1	−1.13045278	1.731869954	0.000396856	0.01799967
KLF5	2.076788399	5.252328688	0.001133446	0.03636955
SPDEF	2.632872658	3.392909267	0.000573362	0.02331316

**Table 3.** Results of correlation analysis

TF	IRGs	Correlation	p value	Regulation
KLF5	S100P	0.706399294	4.6145E-28	postive
SPDEF	S100P	0.629713781	6.07823E-21	postive
KLF5	CETP	−0.504604868	7.99247E-13	negative

To determine whether S100P correlated with tumor immunity, we conducted comprehensive analyses. In the GEPIA database, S100P expression was higher in cancerous tissues than in normal tissues (*p* < 0.01, Figure 4(b)), while the KM curve indicated good predictive capabilities (*p* < 0.01, Figure 4(c)). Higher S100P expression in the TISIDB database also indicated a shorter OS (*p* < 0.01, Figure 4(d)). Therefore, we explored the relationship between S100P expression and common immune inhibitors, finding that S100P expression correlated negatively with CTLA-4 (*p* < 0.01), PDCD1LG2, and PDCD1 (*p* < 0.001; Figure 4(e)). In the TIMER database, S100P expression was positively related to CD4^+^ T cell and macrophage infiltration (*p* < 0.001, Figure 4(f)), with copy number variation analysis confirming that alterations in S100P were associated with CD4 + T cells, B cells, and macrophages (*p* < 0.05, Figure 4(g)).

**Figure 5.**
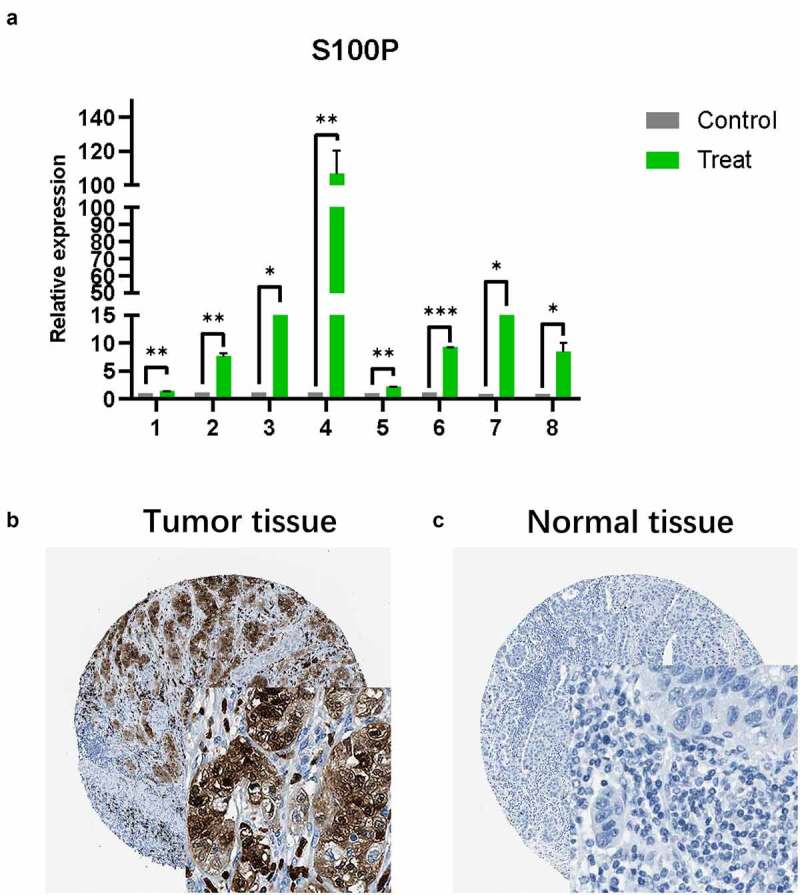
(a) The results of quantitative real-time PCR showed that relative expression level of S100P between tumor and normal tissue. (T1-6: Tumor samples with normal tissues control; C: cell lines with HPDE6-C7 control, C7–8: BxPC-3, SW1990; * P < 0.05; ** P < 0.01; *** P < 0.001.) (b,c) Immunohistochemistry (B: Tumor tissue; C: Normal tissue)

Like the results of transcriptome analysis, qRT-PCR presented the similar trends of S100P (Figure 5(a)), which revealed the S100P involved in the tumorigenesis of pancreatic cancer. Then, we collected immunohistochemical data from the HPA database and found that S100P is highly expressed in tumor tissue at the protein level, this result was consistent with our analysis (Figure 5(b,c)).

### 3.4. Clinical relevance of the prognostic signature

**Figure 6.**
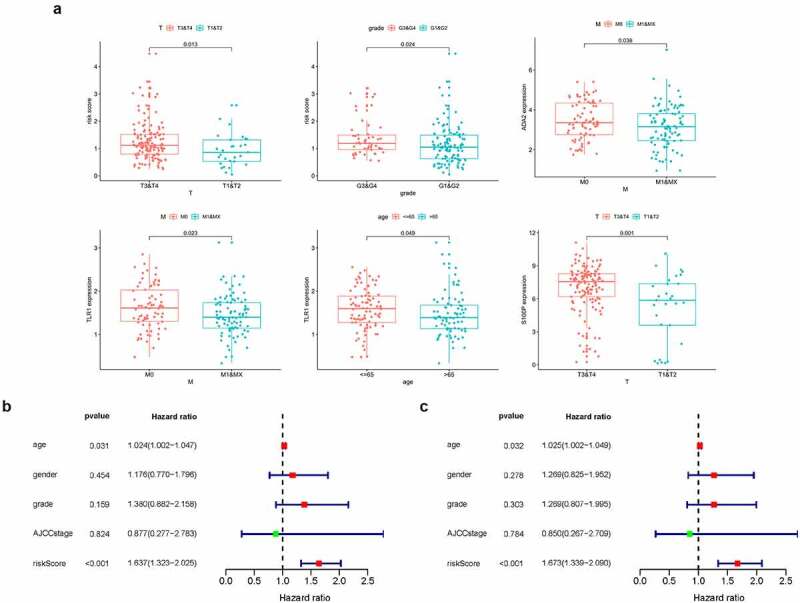
(a) Clinical relevance of the prognostic signature and four IRGs. (b,c) Forest plot of univariate and multivariate regression analyses

Next, we investigated the clinical relevance of the prognostic signature and four IRGs, finding that a higher risk score was related to G3&G4 (*p* = 0.024) and T3&T4 stage (*p* = 0.013), while elevated S100P expression level was significantly in the T3&T4 stage (*p* = 0.001). Both ADA2 and TLR1 displayed lower expression level in M1 stage (*p* = 0.038 and 0.023, respectively) and TLR1 expression was lower in the age ≤ 65 group (*p* = 0.049; Figure 6(a)).

To further verify the independent predictive capability of this signature, we analyzed the correlation between the signature and clinicopathological characteristics such as age, gender, AJCC stage, and grade. Interestingly, univariate and multivariate regression analysis revealed age and risk score as prognostic factors and found that risk score could predict patient prognosis (HR = 1.673, 95% CI = 1.339 − 2.090, *p* < 0.001, Figure 6(b,c)).

### 3.5. Development and validation of a nomogram

**Figure 7.**
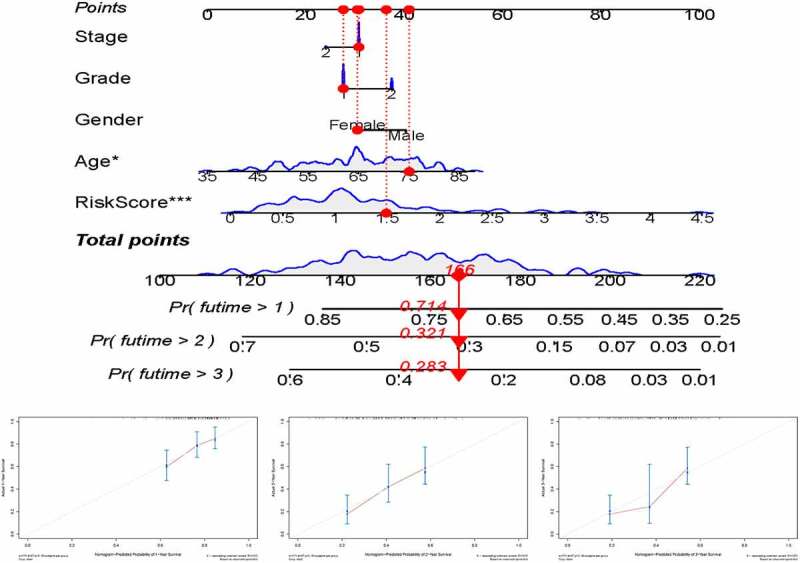
(a) nomogram for predicting OS at 1, 2, and 3 years. (b–d) Calibration curves showing the probability of 1-, 2-, and 3-year OS between the nomogram prediction and practical observation

For preferable clinical application in predicting OS of patients with pancreatic cancer, we constructed a nomogram including clinicopathological characteristics (age, gender, grade, AJCC stage) and the risk score (Figure 7(a)). And the calibration curves showed good agreement between the predicted survival rate and the actual survival rate at 1, 2, and 3 years (Figure 7(c,d)).

### 3.6. Gene set enrichment analysis

Having verified the ability of our prognostic signature to predict OS, we explored the potential mechanism of the prognostic signature by carrying out GSEA. The top five GO terms significantly enriched in the high-risk group like ‘cadherin binding’, ‘sterol biosynthetic process’, ‘mitotic spindle assembly’, ‘apical junction complex’, and ‘cadherin binding involved in cell-cell adhesion’ (*p* < 0.05, Figure S2(a)). KEGG pathway enrichment analysis revealed that the significantly enriched pathways like ‘p53 signaling pathway’, ‘steroid biosynthesis’, ‘tight junction’, ‘pathogenic *Escherichia coli* infection’, and ‘adherens junction’ (*p* < 0.05, Figure S2(b)). these results revealed the mechanism underlying the prognostic signature, in which four IRGs work together in pancreatic cancer.

### 3.7. Correlation analysis of risk score with immune cell infiltration, chemotherapeutics efficacy, and TMB

**Figure 8.**
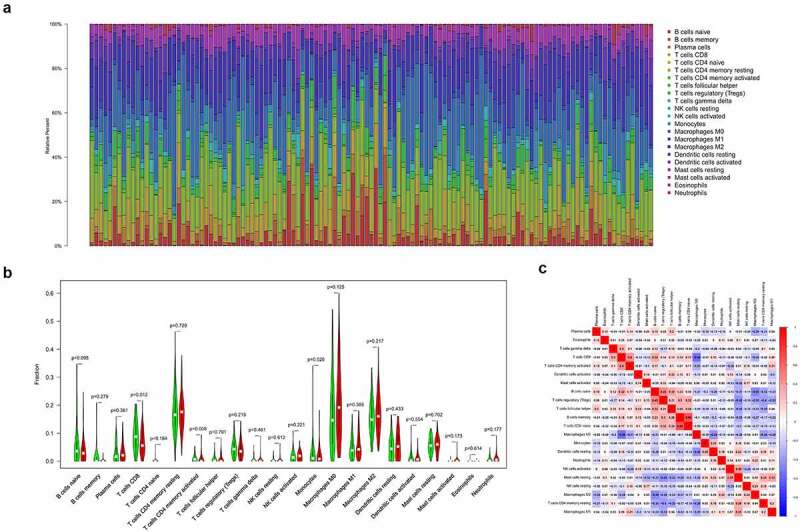
Immune cell infiltration analysis. (a) Distributed histogram of 22 immune cell types. (b) Violin plot comparing immune cell infiltration between the two groups. (c) Correlation heatmap of 22 immune cell types

**Figure 9.**
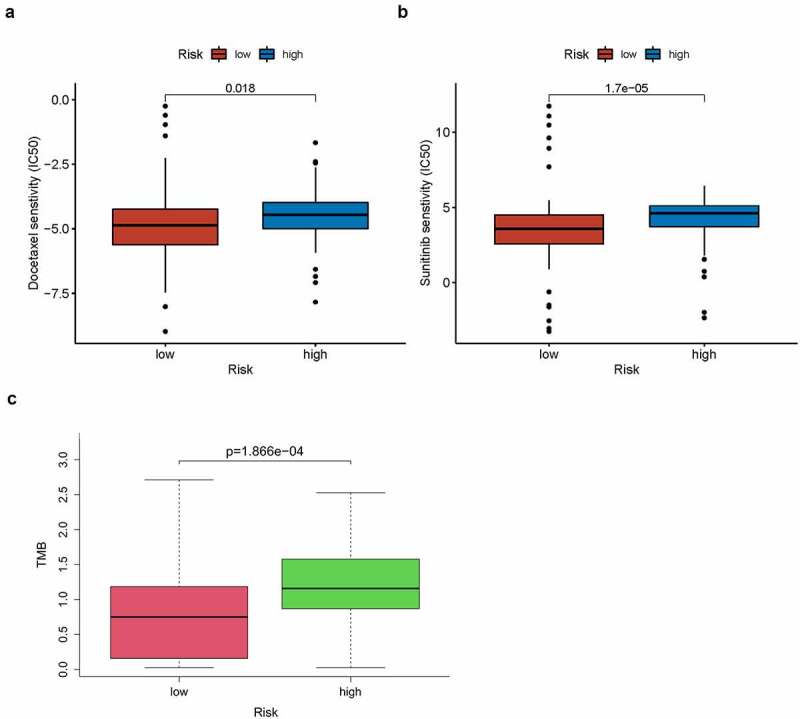
(a,b) The IC50 difference of docetaxel (p = 0.018) and sunitinib (p < 0.001) in high- and low- risk groups. (c) Bar plot of relationship between TMB score and risk score

A total of 123 pancreatic cancer tissue samples were filtered using the CIBERSORT algorithm (*p* < 0.05) and sorted into high- and low-risk groups (*n* = 65 and 58, respectively). The distributed histograms of 22 types of immune cells showed that each sample had different compositions of tumor-infiltrating immune cells (Figure 8(a)). Moreover, the violin plot showed that the high-risk group had lower CD8 + T cell and activated CD4+ memory T cell infiltration in the TIME (*p* < 0.05, Figure 8(b)) and the correlation heatmap showed a weak correlation between different immune cells (Figure 8(c)). The TIMER database, the high TLR1 expression correlated positively with B cells, CD8 + T cells, macrophages, neutrophils, and dendritic cells (*p* < 0.001, Figure S3(a)). The expression of ADA2, also known as CECR1, correlated positively with B cells, CD8 + T cells, CD4 + T cells, macrophages, dendritic cells (*p* < 0.001), and neutrophils (*p* < 0.05; Figure S3(b)), whereas high PTPN6 expression correlated positively with B cells, CD4 + T cells, neutrophils, dendritic cells (*p* < 0.001), and macrophages (*p* < 0.05; Figure S3(c)). Related copy number variation analysis showing the prognostic signature including these three IRGs is shown in (Figure S3(d)). Then, the correlation analysis of risk score with chemotherapeutics efficacy showed the low-risk score was positively related with the higher half IC50 of chemotherapeutic agents Docetaxel (p = 0.018) and Sunitinib (p < 0.001) (Figure 9(a,b)). Finally, TMB was recently described as a novel biomarker that is closely related to immunotherapy; therefore, we collected TMB data for pancreatic cancer from TCGA database. Consequently, we explored the relationship between TMB score and risk score in 151 filtered samples, finding that patients in the high-risk group had higher TMB scores (*p* < 0.001, Figure 9(c)). Therefore, our signature has a certain ability to predict TMB scores.

## 4. Discussion

Pancreatic cancer is associated with a high mortality; therefore, it is important to explore novel clinical biomarkers to improve patient prognosis. Recent studies have shown that immune-related biomarkers are strongly linked to the tumor immune microenvironment (TIME) [32]; therefore, it is essential to investigate new immune-related prognostic markers and explore the mechanism of IRGs-TIME in pancreatic cancer to provide more personalized treatments for pancreatic cancer. In this study, we identified and constructed a novel prognostic signature with good predictive capability and used a bioinformatics approach to explore the potential prognostic mechanisms and their relationship to immune cell infiltration and tumor mutation burden (TMB). Furthermore, we comprehensively analyzed the key immune-related biomarker, S100P, which demonstrated good predictive potential, and verified expression status of S100P in an independent cohort via quantitative real-time PCR and immunohistochemistry.

The novel prognostic signature consisted of four IRGs (ADA2, TLR1, PTPN6, S100P), all of which had independent predictive capabilities. Previous studies have indicated that ADA2, a plasma protein also known as CECR1, is secreted by monocytes and macrophages [33], while Zavialov *et al*. found that ADA2 can increase the proliferation of monocyte-activated CD4 + T cells and stimulate macrophage proliferation [34]. Therefore, ADA2 is expected to act as a biomarker to regulate tumor immune response. TLR1 was the first described mammalian Toll-like receptor and is widely expressed in leukocytes. In addition, TLR1 plays important roles in the immune system, particularly CD8 + T cell regulation [35,36], and the TLR1/2 ligand has been shown to decrease PD-1 expression on antigen-activated CD8 + T cells [37]. Similarly, an anti-tumor vaccine combined with TLR1/2 therapy was found to significantly enhance anti-tumor immunity by decreasing PD-1 expression and inducing antigen-specific CD8 + T cells in a mouse melanoma model [38]. Although the immune mechanisms of ADA2 and TLR1 have been studied in some tumors, they have not yet been studied in pancreatic cancer. Here, we demonstrated that low TLR1 and ADA2 expression play vital roles in pancreatic cancer immunity and can both mediate immune cell infiltration to affect tumor immune status. PTPN6, a non-receptor protein tyrosine phosphatase also known as SHP-1, is an important protein that regulates basic cellular processes and acts as a checkpoint regulator to maintain appropriate immune responses and self-tolerance [39,40]. In addition, PTPN6 has been reported to control cell proliferation and determine the therapeutic potential of somatostatin in pancreatic cancer [41]. In this study, we found that PTPN6 can act as a biomarker that suppresses the tumorigenesis of pancreatic cancer, predict OS, and influence immune cell infiltration to alter tumor status. Therefore, PTPN6 was also suggested as a novel therapeutic and prognostic biomarker. Consequently, we believe that this signature has helped to reveal the role of these proteins in pancreatic cancer and will guide future basic research.

Of the four differentially expressed TFs that we obtained in this study, two (KLF5 and SPDEF) were positively related to S100P, which was a co-DEG in TCGA and GEO databases, suggesting that S100P could be a vital biomarker. S100P is a calcium-binding protein in the S100 family that has been shown to affect pancreatic cancer proliferation, angiogenesis, and metastasis [42,43]. In this study, we analyzed the important functions of S100P in multiple databases using bioinformatic methods, finding that S100P plays important roles in the immune cell infiltration of pancreatic cancer. High S100P expression reduced the infiltration of macrophages and CD4 + T cells, thereby promoting tumor immune escape, and decreased the expression of common immune inhibitors, suggesting that S100P inhibition may improve the expression of treatment targets to improve the efficacy of immunotherapy. We also found that KLF5 regulated the expression of multiple genes; for instance, KLF5 overexpression has been reported to promote proliferation and malignant transformation in a mouse pancreatic ductal adenocarcinoma model [44] and promote tumorigenesis and metastatic potential via the NF-κB signal pathway [45]. However, no studies have yet explored the mechanism between KLF5 and S100P. Here, we found that KLF5 could act as a positive TF with S100P and jointly participate in the malignant biological behavior of pancreatic cancer. More in-depth regulatory mechanisms need be explored to reveal the roles of KLF5 and S100P in order to develop target-inhibitors for individualized clinical treatment.

Prognostic signatures have been studied in many tumors and have been proven to be of high research value. The prognostic signature characterized in this study had a good ability to predict the OS of pancreatic cancer patients; therefore, we performed ROC analysis to further demonstrate its accuracy and efficacy, finding that the signature had moderate accuracy. We also evaluated the clinical relevance of the prognostic signature using Cox regression analysis, revealing that the prognostic signature had independent prognostic capabilities. Furthermore, further comprehend the potential mechanism of the prognostic signature, the GSEA demonstrated that the significantly enriched pathways included ‘p53 signaling pathway’ and ‘steroid biosynthesis’, suggesting that the prognostic signature significantly affects tumor progression.

Immune cells play critical roles in the TIME and thus may affect the response to immunotherapy [46,47]; indeed, several studies have reported that the rate of immune cell infiltration directly affects patient prognosis [48,49]. For instance, Ino *et al*. demonstrated that higher tumor-infiltrating CD4 + T and CD8 + T cell correlated positively with a longer OS [48]. Therefore, we compared immune cell infiltration in the high- and low-risk groups, finding lower CD8 + T cell and activated CD4+ memory T cell infiltration in the TIME of the high-risk group. Unsurprisingly, immune cell infiltration plays an important role in tumor immunity and correlates closely with the OS of patients with pancreatic cancer, consistent with previous studies. Therefore, our prognostic signature appears to identify risk and indicate immune status. We also explored the relationship of the four IRGs in the signature with corresponding immune cells in the TIMER database, finding that all four IRGs can regulate immune cell infiltration and alter efficacy, and thus could be potential new therapeutic targets. In this study, we also proposed that correlation of signature with the sensitivity of common chemotherapeutic drugs, which was expected to an important guide for medication.

Recent studies have shown that TMB can act as a prognostic biomarker and affect tumor response to immunotherapy [50]. Patients with higher TMB harbor cancer cells with more mutations that differ more obviously to normal cells, and are therefore more easily detected by immune cells and respond better to immunotherapy. A recent clinical trial reported that a high TMB can predict the response of patients receiving pembrolizumab [51], and TMB has become an independent prognostic factor in many cancers, including lung adenocarcinoma [52], colorectal cancer [53], and gliomas [54]. In this study, we analyzed the TMB score of each patient with pancreatic cancer. Further analysis of 151 patients with TMB scores indicated that the high-risk group had higher TMB scores than the low-risk group. Therefore, risk scores may reflect TMB levels and have ability to predict immune responses and the efficacy of immunotherapy. In this study, the patients with a high TMB score and a high-risk score as indicated by our prognostic signature may benefit from immunotherapy and that the signature could guide patient diagnosis and treatment.

Despite these findings, our study has some limitations. Firstly, all the data retrospectively analyzed in our study were obtained from public databases; therefore, selection bias is inevitable and further large prospective cohort studies must be implemented to confirm the efficacy of our prognostic signature. Then, due to the different sequencing methods in TCGA and GEO database, Standardization and subsequent processes were difficult to be unified in our study, so there was no validation set from the GEO database. finally, although we analyzed the potential molecular mechanism of our prognostic signature, the effectiveness of the TIME and TMB, it needs to further be verified in pancreatic cancer *in vivo* and *in vitro*.

## 5. Conclusion

In this study, we constructed an immune-related prognostic signature based on four immune-related genes (ADA2, TLR1, PTPN6, and S100P) that displayed good predictive ability for overall survival and analyzed the infiltration of corresponding immune cells in patients with pancreatic cancer. Furthermore, we comprehensively analyzed the key immune-related biomarker, S100P, which demonstrated good predictive potential. Promisingly, this prognostic signature provides a new perspective to explore biomarkers for future personalized immunotherapies for pancreatic cancer.

## Supplementary Material

Supplemental Material

## Data Availability

The data used to support the results of this study can be obtained from The Cancer Genome Atlas (TCGA, https://cancergenome.nih.gov/), GEO database (https://www.ncbi.nlm.nih.gov/geo/), Immport database (https://immport.niaid.nih.gov), TISIDB database (http://cis.hku.hk/TISIDB/), TIMER database (http://timer.cistrome.org/), HPA database (https://www.proteinatlas.org/).
