# Supplementary material for: Up-regulation of S100P predicts the poor long-term survival and construction of prognostic signature for survival and immunotherapy in patients with pancreatic cancer
Source: Bioengineered. 2021 Oct 27;12(1):9006–20. doi: 10.1080/21655979.2021.1992331 (PMC8806945; doi:10.1080/21655979.2021.1992331)
Supplement: Supplemental Material [file KBIE_A_1992331_SM9871.docx]

Supplementary materials

**Title:**

Up-regulation of S100P predicts the poor prognosis and construction of prognostic signature for survival and immunotherapy in patients with pancreatic cancer

**Running Title:**

Novel immune-related biomarkers in pancreatic cancer

**Supplementary FigureS1**


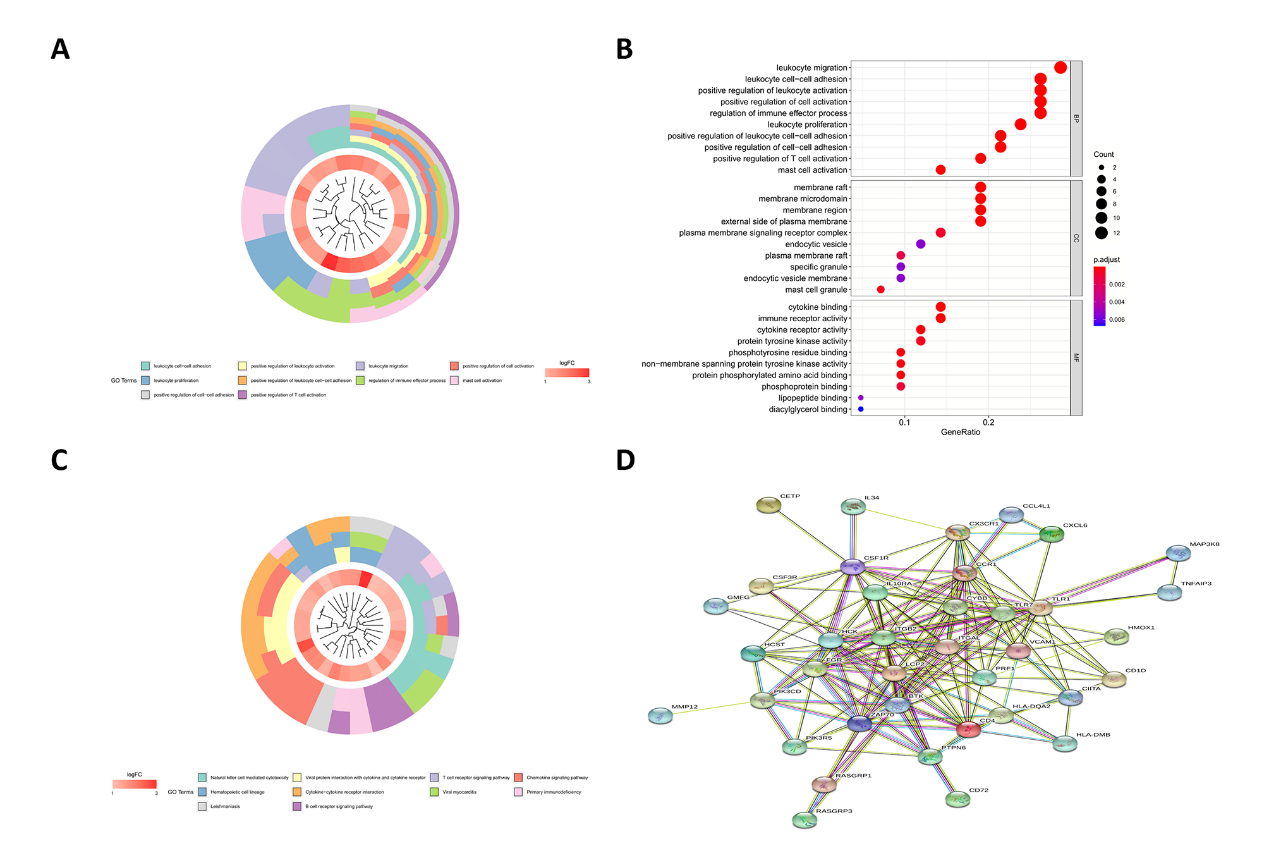


Supplementary FigureS1. Functional enrichment analysis and PPI network. A-B GO enrichment analysis. C KEGG pathway enrichment analysis. D PPI network.

**Supplementary FigureS2**


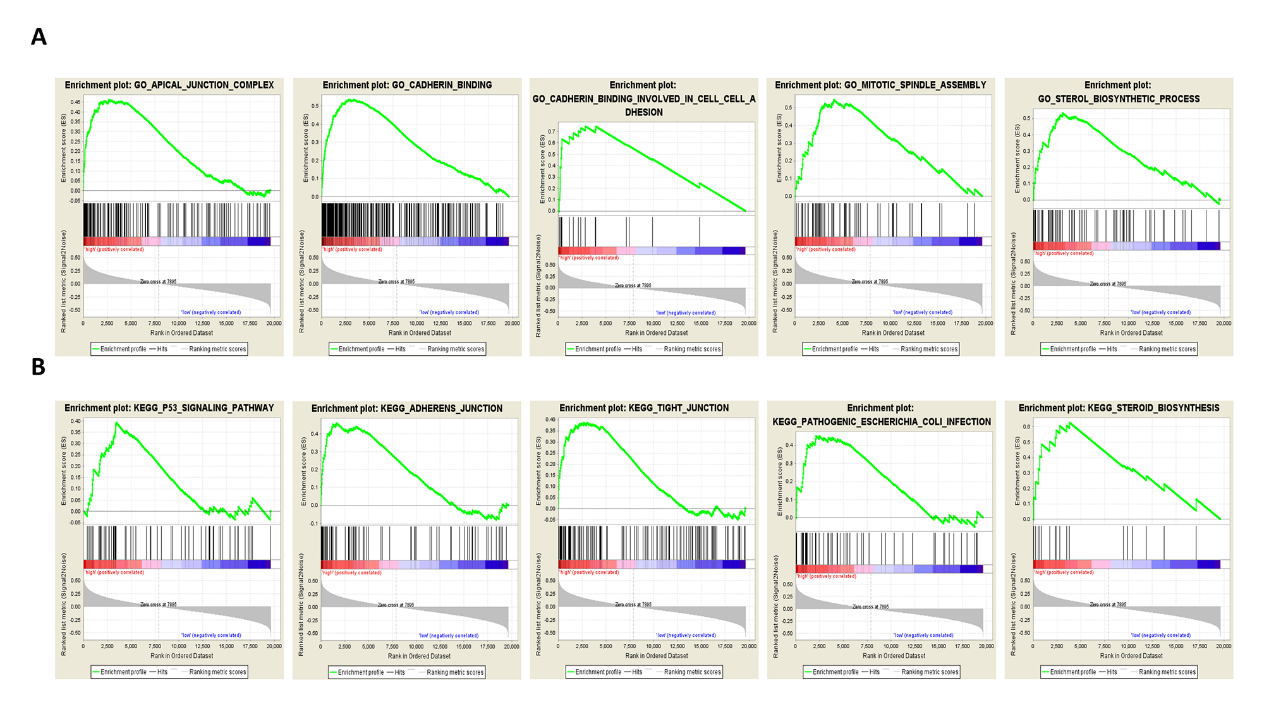


Supplementary FigureS2**.** Functional enrichment analysis. **A** Top five enriched GO terms. **B** Significantly enriched KEGG pathways.

**Supplementary FigureS3**


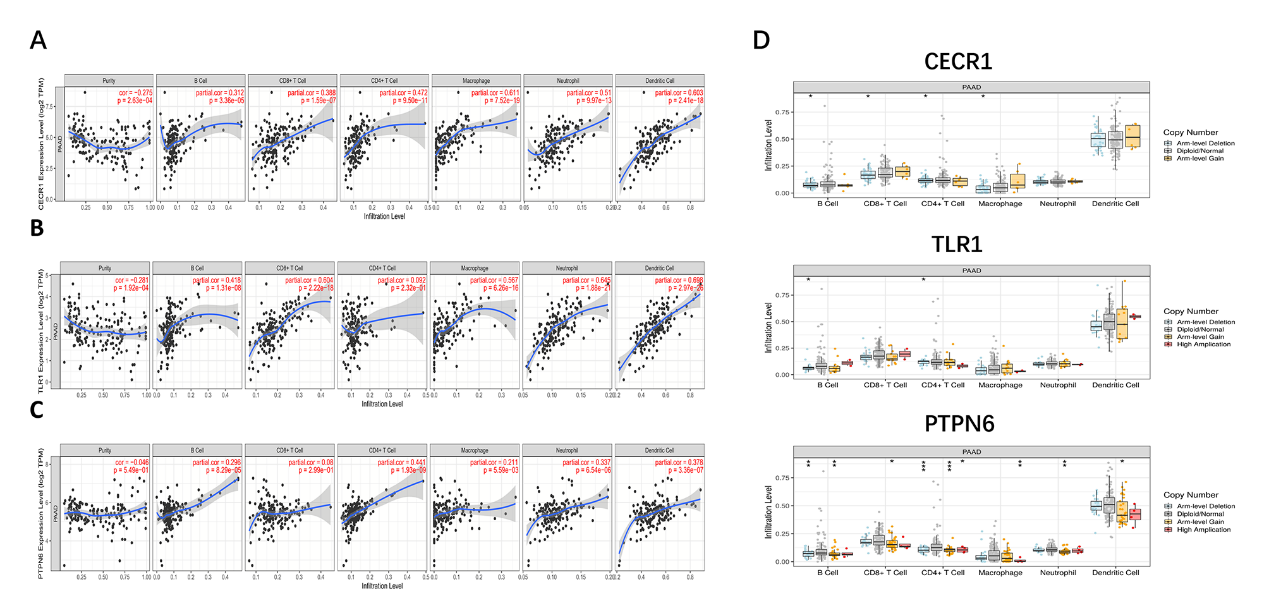


Supplementary FigureS3**.** Correlation analysis in the TIMER database. **A-C** Correlation of immune cell infiltration with CECR1, TLR1, and PTPN6. **D** Corresponding copy number variation analysis of the prognostic signature.

# sTable 1. Comparison of long-term survival between patients with high- and low-risk score

| **Cohorts** | **Survival Time** | **Low-risk (n=89)** | **High-risk (n=88)** | ***P* value** |
| --- | --- | --- | --- | --- |
| **Total cohort**  **(n=177)** | 1-year OS rate (%) | 78.4 | 71.4 | **<0.01** |
|  | 2-year OS rate (%) | 49.1 | 31.0 |  |
|  | 3-year OS rate (%) | 43.0 | 15.1 |  |
|  | Median OS (95% CI), months | 48.8 (38.4-59.1) | 25.2 (19.0-31.4) |  |
| Abbreviations: OS, overall survival; CI, confidence interval | | | | |
